# How nostalgic taste on the screen stimulates the consumption of time-honoured restaurants: The mediation role of parasocial interaction

**DOI:** 10.3389/fpsyg.2022.1062315

**Published:** 2022-12-07

**Authors:** Jian Yang, Jianle Tang, Lingmin Zhang

**Affiliations:** ^1^School of Journalism and Communication, Guangzhou University, Guangzhou, China; ^2^School of Public Administration, Guangzhou University, Guangzhou, China

**Keywords:** nostalgia, parasocial interaction, emotional involvement, credibility, consumer behaviour, blogger, time-honoured restaurant

## Abstract

As a unique cultural asset, time-honoured restaurants carry the crystallisation of traditional cuisine and the spirit of artisanship with an inestimable value. Nostalgia is a characteristic element of time-honoured restaurants and the central theme of their online marketing. However, few studies have examined the effect of nostalgia evoked in bloggers’ content on consumer behaviour in the context of time-honoured restaurants. To fill this gap, this study employed the SOR model as an underpinning theory and selected parasocial interaction among bloggers and viewers as a mediation to examine how the nostalgic taste on the screen affected behavioural intention towards time-honoured restaurants. Emotional involvement and credibility were also added as variables to enrich the research framework from cognitive and affective perspectives. A PLS-SEM approach was used to analyse the research models, including evaluating the measurement and structure models. The result, tested by the online survey data from 319 valid responses, demonstrated that nostalgia evoked in bloggers’ content can directly facilitate parasocial interaction or indirectly through credibility and emotional involvement, finally resulting in behavioural intention. The fully mediating role of parasocial interaction between emotional involvement and behavioural intention was also confirmed. The findings of this study offer theoretical and practical implications, highlighting the critical roles of nostalgia and parasocial interaction in the mechanism that online stimulus influences realistic behavioural intention, and therefore exploring a way forward for time-honoured restaurant marketing that fits in with the online media age.

## Introduction

Time-honoured restaurants are deemed an important component of the national culinary heritage, cultural resources and social assets (Song et al., 2021). In addition to their unique cooking techniques, time-honoured restaurants are becoming increasingly famous as a result of their culinary skills passed down from generation to generation (Song and Kim, 2021). Time-honoured restaurants which serve both non-commercial and commercial functions not only have the inestimable spiritual value that play the role of heirs and hosts of national culture and builders of local image; they also make substantial economic contributions to the restaurant industry and tourism (Shang and Chen, 2016; Song and Kim, 2021; Zhang et al., 2021). Time-honoured restaurants have become a promising and increasingly prominent segment of the food service industry (Balogh et al., 2016). However, as the hospitality industry undergoes intense competition, certain time-honoured restaurants are reported to be in decline, suffering long-term losses and even facing bankruptcy (Chen et al., 2020). Compared with emerging restaurants, time-honoured restaurant catering enterprises lack vitality and strong development momentum; additionally, revitalising these restaurants continues to face serious challenges (Zhang et al., 2021).

With the emergence of online media and leisure consumption, many people watch leisure content on online media, which offers the possibility of nostalgic consumption. When respect to online media content, bloggers are an essential group who act as content generators; they deliver content to viewers and can influence and lead viewers’ decisions (Uzunoğlu and Misci Kip, 2014). For example, bloggers who generate content about time-honoured restaurants share their introductions, experiences and opinions about the restaurants, even other stories about themselves in several forms such as articles, graphics and videos; this group is believed to be a tool for consumers to choose which restaurant suits their needs (Puspita and Hendrayati, 2020). Considering the plight of the survival of time-honoured restaurants, this study believes in the feasibility of exploring online marketing strategies for them. As a strategy of online marketing, however, bloggers’ promotion has not received sufficient attention in the research on time-honoured restaurants.

Research related to time-honoured brands is often linked to the theme of nostalgia (Zhou et al., 2013; Gu et al., 2021; Song et al., 2021). Song et al. (2021) have explored how nostalgia triggers (as antecedents) lead to consumers’ revisiting intention (as the outcome) by influencing nostalgic experiences, thereby affirming its vital role in time-honoured restaurant marketing. Nostalgia can be evoked by various paths such as scent, food, music and specific events or experiences (Holak and Havlena, 1992; Havlena and Holak, 1996; Wildschut et al., 2006; Reid et al., 2015). In reality, bloggers promoting time-honoured restaurants often evoke nostalgia as a strategy. However, research scarcely focuses on nostalgia triggered by the content on online media instead of the nostalgic experience on site in the context of consumer behaviour.

In the context of online social media, traditional word-of-mouth (WOM) has moved online to become electronic word-of-mouth (e-WOM) (Dogra and Kale, 2020). This factor influencing consumer behaviour may be affected by online content such as bloggers’ personal stories, ideas, reviews, opinions, feelings and emotions (Sokolova and Kefi, 2020). A special interaction called, parasocial interaction (the illusion of a face-to-face relationship with a media personality), occurs between bloggers and viewers (Horton and Wohl, 1956). Its value on social media as a marketing strategy for promotion has also been explored (Rasmussen, 2018). However, research on parasocial interaction in the restaurant context is extremely limited, especially the time-honoured restaurant. In addition, the effect of nostalgia evoked by blogger’s content on the parasocial interaction between the blogger and viewers is unclear.

As an important indicator of customers’ future behaviours (Lai and Chen, 2011), behavioural intention can be used to test the effectiveness of marketing. Therefore, a necessity arises to explore how nostalgia evoked by the blogger’s content ultimately affects viewers’ behavioural intentions by influencing parasocial interaction between viewers and the blogger. To fully complete this process with cognitive and affective factors, this study also includes two other variables, namely, the credibility of the blogger and viewers’ emotional involvement with the blogger. From a cross-disciplinary perspective, our study aims to develop a comprehensive model, establishing links between nostalgia, parasocial interaction, credibility, emotional involvement and behavioural intention in the context of time-honoured restaurants. The theory of planned behaviour (TPB) and theory of reasoned action (TRA) have been widely used in online consumer behaviour research. However, for the current study, these theoretical backgrounds reflect only part of the research framework and do not pay sufficient attention to external environmental factors (Huang et al., 2017). Therefore, the stimuli-organism-response (SOR) model proposed by Mehrabian and Russell (1974) was employed as a theoretical underpinning in this study. It could explain the process that the environmental stimuli lead to an individual internal psychological state, which then results in individual’s consumption behaviour.

Overall, in the online media era, bloggers can be considered digital influencers and potential opinion leaders (Uzunoğlu and Misci Kip, 2014) and can even become a benchmark for people in considering whether or not a restaurant is worth visiting (Syahbani and Widodo, 2017). Therefore, the current study aims to explore the nostalgic triggers of bloggers’ content marketing in the sustainable development of time-honoured restaurants, providing theoretical and practical contributions in this context. Specifically, based on the SOR model, the current study aims (1) to investigate how nostalgia as a stimulus affects parasocial interaction, credibility and emotional involvement and further influences behavioural intention as a response; (2) to examine the relationship between credibility and parasocial interaction, emotional involvement and parasocial interaction, respectively and (3) to examine whether nostalgia evoked by bloggers’ content directly influences viewers’ behavioural intention. The findings of the study provide both theoretical and practical implications for the online promotion of time-honoured restaurants, the operation of bloggers, and the development of policies for the sustainable development of time-honoured restaurants.

The remainder of this study is organised as follows. Section 2 reviews the literature on nostalgia, parasocial interaction, credibility, emotional involvement, and behavioural intention and proposes nine research hypotheses. The methodology for the research, which includes data collection, research instrument, and the analytical procedure, is described in detail in section 3. The research results are reported in section 4 and discussed in section 5. And in section 6, the research findings are summarised and concluded, and the theoretical and practical implications are provided. Finally, section 7 addresses the limitations of this research and recommendations for future study.

## Literature review

### Nostalgia

Davis (1979) defined nostalgia as ‘a positively toned evocation of a lived past’ and found that many positive sentiments are expressed with regard to it. Holbrook and Schindler (1991) considered nostalgia as a preference (general liking, positive attitude or favourable effect) towards objects (people, places or things) that were more common (popular, fashionable or widely circulated) when one was younger (in early adulthood, in adolescence, in childhood or even before birth). From a positive and functional perspective, nostalgia conceptually provided a new vision for marketing. Most previous studies on nostalgia marketing have focused on nostalgia triggered by the brand or product itself and confirmed its positive role (Kessous et al., 2015; Olsen et al., 2021; Youn and Dodoo, 2021). However, research has rarely paid attention to nostalgia evoked in bloggers’ content.

In this study, the nostalgia evoked in the content about time-honoured restaurants consists of two parts. On the one hand, nostalgia triggers related to food, environment, atmosphere, event, staff and service play a positive role in restaurant marketing (Hwang and Hyun, 2013; Wen et al., 2019; Gu et al., 2021). These nostalgia triggers can be shown in the blogger’s content in the blogger’s own way; in other words, nostalgia can be evoked through a reduction of the time-honoured restaurant itself in the blogger’s content. On the other hand, part of what evokes nostalgia may be factors related to the blogger. Nostalgia was conceptualised as a positively valanced complex feeling, emotion or mood produced by a reflection on things (objects, persons, experiences or ideas) associated with the past (Holak and Havlena, 1998). Specifically concerning the content about time-honoured restaurants, the blogger may introduce the restaurant by telling a story that relates to the good times from the past, such as what happened to the restaurant these years, what the city where the restaurant is located looked like before or how happy individuals were when enjoying delicious food with families and friends at a younger age. When dining in the restaurant, other customers may share similar sentiments or social ethos with the blogger, which could act as a stimulus to the viewers (Gu et al., 2021). Therefore, people are likely led into a nostalgic state when viewing the content, especially those who grew up in the city where the restaurant is located but are now far from there or those who have left good memories there.

Stimulus can be defined as an influence that arouses the individuals, which is a factor that affects the internal states (Eroglu et al., 2001). Nostalgia has been used as a stimulus in the SOR model in previous research (Cho et al., 2019; Slavich et al., 2019; Cho et al., 2020; Chang et al., 2022). For example, a study argued that objects presented through the media can act as a stimulus to evoke nostalgia among sport fans as they associate specific objects with sport-related special moments from their past (Cho et al., 2020). Therefore, nostalgia in the content about time-honoured restaurants is considered to have a similar effect, that is, the stimulus (S) in the SOR model refers to nostalgia evoked by the blogger’s content about a time-honoured restaurant.

### Parasocial interaction

The term ‘parasocial interaction’ was originally coined by Horton and Wohl (1956), which describes the illusion of a face-to-face relationship with a media personality. Parasocial interaction is regarded as a psychological phenomenon (Giles, 2002), a faux sense of mutual awareness (Dibble et al., 2016) and viewers’ feelings of interpersonal involvement with bloggers *via* social media (Pornsakulvanich and Tongnok, 2022). Although parasocial interaction theory was originally employed in the context of traditional media such as television and radio, recent studies have also applied it to online media such as social and new media (Frederick et al., 2012; Penttinen et al., 2022). In the context of bloggers, parasocial interaction resembles a psychological perception of viewers in that they feel as if the blogger is a friend, and they are allowed to seek advice and psychological support from the latter.

People can conveniently communicate, connect, interact and build relationships with bloggers on social media platforms. The content generated by bloggers can strengthen social connectedness between bloggers and viewers, which somewhat promotes parasocial interaction (Pornsakulvanich and Tongnok, 2022). Based on parasocial interaction theory, the parasocial relationship formed between blogger and viewers was considered a socio-emotional connection that people develop with media figures (Hoffner and Bond, 2022). As a predominantly positive, self-relevant and social emotion that plays key psychological functions, nostalgia can foster social connectedness (Sedikides et al., 2008; Wildschut et al., 2010) and people’s liking (Wen-Shin et al., 2011). In addition, Wulf et al. (2018) analysed the existing literature and argued that a conceptual overlap existed between nostalgic and hedonic entertainment experiences. Additionally, a recent study found that parasocial interaction is positively related to hedonic enjoyment such as the experience of strong positive emotions (Stein et al., 2022). When nostalgia evoked in the content about time-honoured restaurant reminds the viewer of ‘good old days’ memories, he/she would be immersed in positive emotions. Nostalgia serves as a stimulus and provides an imaginary scene for the viewer to enjoy such a psychological process, which associates positive emotions with the blogger and further fosters parasocial interaction.

Considering the above discussion about nostalgia and parasocial interaction, we propose the following hypothesis:

*H1*: Nostalgia has a positive influence on parasocial interaction.

### Credibility and emotional involvement

Source credibility was defined as the degree to which an individual perceives the media source as real (Austin and Dong, 1994). In the online environment, bloggers are regarded as the source, and blogger credibility refers to viewers’ perception of the expertise and trustworthiness of the blogger (Cosenza et al., 2015) and plays an important role in media marketing.

In the context of brand communication about nostalgia, nostalgia is utilised to evoke emotion among consumers and gives brands a sense of credibility, authenticity, durability and quality as well as emotional bonding (Kessous and Roux, 2013). For example, a brand that links the nostalgia can create a reassuring effect (Grębosz and Pointet, 2015). By evoking people’s personal memories, a nostalgic spokes-character stimulates these people to associate brands with characters they have trusted from childhood (Neeley et al., 2000; Sheehan, 2020), and the trust may extend to the nostalgic spokes-character. Garretson and Niedrich (2004) found that nostalgia elicited by characters will favourably affect spokes-character trust. On the relationship between trust and credibility, Doney and Cannon (1997) defined trust as the perceived credibility and benevolence of a target of trust. In our study, the target of trust refers to bloggers who have produced content about time-honoured restaurants. Apparently, nostalgia can confer a positive trait to the blogger, thus making viewers feel that the blogger is credible.

Credibility reflects the reliability, correctness, objectivity and value as perceived by the readers of the information sources and content posted by bloggers on their blogs (Maggiore et al., 2022). In the content about time-honoured restaurants, the blogger somewhat acts as a narrator. He/she may not only introduce the time-honoured restaurant but also talk about something other than the restaurant such as his/her real experience in the past, which may induce authentic and credible feelings among audiences. Thus, that particular narrative serves as the information source enhancing the credibility of the blogger.

In the context of celebrity, celebrity involvement describes viewers’ tendency to develop a heightened affection and attachment to a celebrity (Lee et al., 2008), which reflects a psychological and emotional state. Nostalgia can facilitate the degree of involvement with things related to the film, such as the location, characters, props, writer, scenes and featured scenery (Kim et al., 2017). A film with nostalgic features can evoke audiences to experience emotional involvement with things associated with the film (Kim et al., 2017). The content about time-honoured restaurants generated by bloggers may have a film-like function to entertain viewers, and the same can be done to foster their emotional involvement. The positive feeling such as pleasure associated with nostalgia offers an emotional value (Sullivan et al., 2012), and emotional involvement is one of the value types in the context of consumer behaviour. Viewers will gain additional involvement with the blogger or his/her content including emotional involvement when the blogger’s content is relatable to their own experience (Halim and Kiatkawsin, 2021). For example, the time-honoured restaurant in the blogger’s content may be an unforgettable part of his/her past life; many sweet memories are related to the restaurant, and even he/she finds a common experience with the blogger at a certain moment, which may predict their emotional involvement.

According to the explanation of the SOR model by Eroglu et al. (2001), organism is represented by cognitive and affective states and processes. In our study, credibility belongs to a cognitive state (Metzger, 2007), whereas emotional involvement belongs to an affective state, that is, both credibility and emotional involvement are organisms in the SOR model.

Considering the above discussion about credibility, emotional involvement and nostalgia, we propose the following hypothesis:

*H2*: Nostalgia has a positive influence on credibility.

*H3*: Nostalgia has a positive influence on emotional involvement.

Previous studies have investigated how credibility facilitates the parasocial interaction, affirming the important role of credibility. For example, trustworthiness and expertise positively influence parasocial interaction in the tourism context (Zhang et al., 2020; Yılmazdoğan et al., 2021). Endorser’s credibility was found to positively influence followers’ parasocial relationship (Zheng et al., 2022). Regarding bloggers’ content, viewers will have parasocial interaction if the information is credible (Nurul and Muhammad, 2020). Bloggers’ credibility could lead the blogger’s content viewers closer to the blogger, which increases the likelihood of strengthening their parasocial interaction and results in positive marketing outcomes. In other words, the credibility of bloggers is an important prerequisite for a high level of parasocial interaction. Fake news, low-credible source or the blogger’s untrustworthy performance neither offers useful help to viewers nor facilitates viewers’ trust in the blogger, thereby anticipating termination of their interaction. During the process of absorption into a story that entails imagery, affect and attentional focus (Rubin et al., 2003), viewers become emotionally and psychologically involved in both the content and with the blogger in the content; they feel like they are the person in that narrative world, thus further leading the parasocial interaction with the blogger (Rojek, 2015). If the content viewer becomes emotionally involved with either the content or the blogger, then, he/she may easily generate attachment with the blogger including psychological reflection or behavioural response. For example, a blogger who satisfies a viewer seeking for help and showing a profound emotion about the content, such as when the blogger introduces a time-honoured restaurant through a story full of emotions, the viewer may feel that he/she is a part of the story and immersed in the story (Tercia et al., 2022). To take it a step further, at that moment, the viewer’s high level of emotional involvement may tend to increase the intimacy with the blogger, even creating a potential motivation to transform the psychological process into a more behaviourally oriented state.

In the SOR model, organism refers to the internal states of perceptions, feelings and thinking exercises (Bagozzi, 1986), which represent the internal emotion and psychological process after the external stimulus (Pandita et al., 2021). Parasocial interaction was conceptualised as the interpersonal involvement of the media user with a media persona, which includes seeking guidance from a media persona, seeing media personalities as friends, imagining being part of a favourite programme’s social world and desiring to meet media performers (Rubin et al., 1985). Moreover, it was considered a shorthand for cognitive and affective reactions (Schiappa et al., 2005). In light of the various properties of parasocial interaction, we consider it a variable between organism (O) and response (R) in the SOR model in the current study.

Considering the above discussion about credibility, emotional involvement and parasocial interaction, we propose the following hypothesis:

*H4*: Credibility has a positive influence on parasocial interaction.

*H5*: Emotional involvement has a positive influence on parasocial interaction.

### Behavioural intention

Behavioural intention is considered an affirmed likelihood to engage in a certain behaviour, which is a significant indicator of customer’s future behaviours (Lai and Chen, 2011). Thus, this positive antecedent could encourage people to behave in a way that benefits the time-honoured restaurant. As important information for restaurant operation (Su, 2011), behavioural intention in the current study refers to viewers’ intention to consume in the time-honoured restaurant mentioned in the bloggers’ content, to recommend this restaurant to families and friends and to leave a relatively positive comment on this restaurant.

In the context of marketing based on blogger’s content, credibility of bloggers could encourage potential consumers to generate positive behavioural intentions (Chaovalit, 2014). Source credibility has positive effects on consumers’ purchase intention (Weismueller et al., 2021). Compared with less credible sources, highly credible sources generate a more positive attitude and induce more behavioural compliance (Chu and Kamal, 2008). To enhance consumers’ positive behavioural intention, the credibility of bloggers may be an important antecedent. An empirical study identified that the more emotional involvement audience develops by viewing the TV drama, the greater the likelihood that they will visit the film tourism destination (Kim, 2011). Similarly, when viewers generate a higher level of emotional involvement with the blogger in the content about time-honoured restaurants, they would have an increased likelihood of consumption behaviour. That is, emotional involvement has the capacity to act as a predictor role of behavioural intention. Parasocial interaction is demonstrated to positively influence consumers’ attitudes and behavioural intentions (Lee and Watkins, 2016; Hwang and Zhang, 2018; Gong, 2020). Moreover, Tian and Hoffner (2010) argued that parasocial interactions can motivate people in their efforts to change themselves to become more like media characters. Thus, the following can be inferred: the higher level of parasocial interaction between the blogger and viewers, the more likely viewers will form an intention to consume at the time-honoured restaurants that the blogger likes. In addition, within the context of restaurant marketing, triggered nostalgia can be a powerful stimulus in influencing consumer behaviour and was indicated to have positive impacts on behavioural intention and willingness to be engaged in word-of-mouth behaviours (Chen et al., 2014, 2020). Considering the desire to experience the nostalgic atmosphere and food, the nostalgic stimuli that appear in the blogger’s content about a time-honoured restaurant can motivate viewers to recommend the restaurant to others, to visit it and to leave a positive comment about it.

In the SOR model, response is considered as the dependent variable, which was affected by stimulus as an independent variable and organism as mediator (Goi et al., 2014). Behavioural intention is affected by the above variables discussed and was used to be the response (R) in the SOR model in our study, which is consistent with some previous studies in the context of restaurant (Jang and Namkung, 2009; Namkung and Jang, 2010; Gineikienė, 2013; Yan et al., 2018; Rajput and Gahfoor, 2020).

Considering all the above variables that may influence behavioural intention, we propose the following hypothesis:

*H6*: Credibility has a positive influence on behavioural intention.

*H7*: Emotional involvement has a positive influence on behavioural intention.

*H8*: Parasocial interaction has a positive influence on behavioural intention.

*H9*: Nostalgia has a positive influence on behavioural intention.

**Figure 1:**
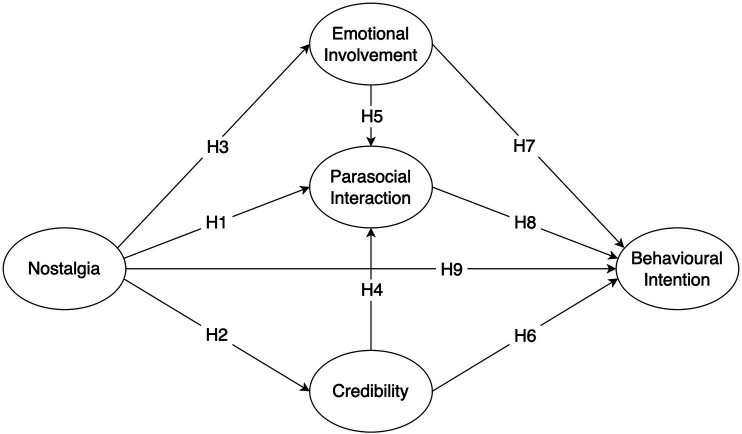
Theoretical Framework.

Based on Hypotheses 1 to 9 as proposed above, Figure 1 presents the theoretical framework of this study.

## Methodology

### Data collection

As a Cantonese cuisine centre, Guangzhou has been well recognised as a ‘place for eating’ since the 1980s. Guangzhou’s time-honoured restaurants use nostalgic commercialisation for promoting their establishments, thus forming a considerable local market. Many existing studies related to food tourism and culinary nostalgia were in the context of Guangzhou (Klein, 2008; Ding et al., 2022). Therefore, our target population is the individuals who have watched the blogger’s content about Guangzhou’s time-honoured restaurants. Specifically, the research was conducted among individuals who (1) have browsed the contents about Guangzhou’s time-honoured restaurants recommended by bloggers and (2) could recall the most impressive name of the time-honoured restaurant.

A convenience sampling method was applied to data collection. Online surveys have been identified as a valid method in studies related to parasocial interaction (Haobin Ye et al., 2020). From July 1st to August 31st 2022, the link to the online survey was initially distributed through the researchers’ personal social media networks. Following completion of the survey, respondents were encouraged to share the survey link with their friends and family members. A two-question screening process was used to identify whether a participant belonged to the study. Survey participants would be eliminated if they did not browse the blogger’s content about Guangzhou’s time-honoured restaurants or failed to provide a specific name of the restaurant. Furthermore, participants who provided the name of a time-honoured restaurant that had closed down on the questionnaire were considered inappropriate participants. Subsequently, participants were asked to choose their opinions based on their most memorable restaurant and relevant blogger.

### Research instrument

The questionnaire included general demographics (e.g., gender, age, education, income, etc.), frequency of reading the bloggers’ social network, perceived nostalgia for the blogger’s content, parasocial interaction, emotional involvement and credibility of the favourite blogger and behavioural intention to the time-honoured restaurant. To evaluate how consumers’ perceived nostalgia from bloggers’ content about time-honoured restaurants influences behavioural intention, the questionnaire with 32 items was designed to measure five constructs in the research framework. The measurement scales used in this study were established from existing research and have been proven to be reliable and valid. Back-translation was used as all questionnaire items were originally written in English, ensuring that the Chinese and English versions of the questionnaire were equivalent (Brislin, 1970). Specifically, nostalgia was measured using the scale adapted from Pascal et al. (2002). An example item could be ‘When watching this blogger’s content about time-honoured restaurants, I think it reminds me of good times in the past’. Parasocial interaction was measured using the scale adapted from previous studies (Rubin et al., 1985; Lee and Watkins, 2016). An example item could be ‘If this blogger appears on another platform, I will watch his/her content’. Credibility was measured using the scale adapted from Cosenza et al. (2015) and Mainolfi et al. (2021). An example item could be ‘I think this blogger is informed about time-honoured restaurants’. Emotional involvement was measured using the scale adapted from Kim et al. (2018). An example item could be ‘When watching this blogger’s content about time-honoured restaurants, I felt that I was part of the story’. Behavioural intention was measured using the scale adapted from Liu et al. (2005), Lin (2006) and Gu et al. (2021). An example item could be ‘I plan to go to this time-honoured restaurant for consumption’. A seven-point Likert scale was used in this study to reflect the respondent’s feedback from 7 (‘strongly agree’) to 1 (‘strongly disagree’). Table 1 presents all specific items.

**Table 1:** Study constructs and measurement items.

Constructs and measurement items		Previous study
Nostalgia (NOS)		
NOS1	When watching this blogger’s content about time-honoured restaurants, I think it reminds me of the past.	Pascal et al. (2002)
NOS2	When watching this blogger’s content about time-honoured restaurants, I think it helps me recall pleasant memories.	
NOS3	When watching this blogger’s content about time-honoured restaurants, I think it makes me feel nostalgic.	
NOS4	When watching this blogger’s content about time-honoured restaurants, I think it makes me reminisce about a previous time.	
NOS5	When watching this blogger’s content about time-honoured restaurants, I think it evokes fond memories.	
NOS6	When watching this blogger’s content about time-honoured restaurants, I think it is a pleasant reminder of the past.	
NOS7	When watching this blogger’s content about time-honoured restaurants, I think it brings back memories of good times from the past.	
NOS8	When watching this blogger’s content about time-honoured restaurants, I think it reminds me of the good old days.	
NOS9	When watching this blogger’s content about time-honoured restaurants, I think it reminds me of good times in the past.	
Parasocial Interaction (PSI)		
PSI1	I am highly looking forward to watching more of this blogger’s content on the Internet platform.	Rubin et al. (1985), Lee and Watkins (2016)
PSI2	If this blogger appears on another platform, I will watch his/her content.	
PSI3	When I was watching this blogger, I felt as if I could fit into his/her group.	
PSI4	When I was watching this blogger, I felt like I was on board with him/her.	
PSI5	I would be glad to meet this blogger in person.	
PSI6	When this blogger shows me how he/she feels about the time-honoured restaurant, it helps me make up my own mind about this time-honoured restaurant.	
Credibility (CRED)		
CRED1	I think this blogger is informed about time-honoured restaurants.	Cosenza et al. (2015), Mainolfi et al. (2021)
CRED2	I think this blogger has a relatively sincere interest in time-honoured restaurants.	
CRED3	I highly respect this blogger.	
CRED4	This blogger highly respects my opinion/evaluation.	
CRED5	I expect this blogger to reveal all conflicts of interest.	
CRED6	Once I established a relationship with this blogger, I never questioned his or her integrity.	
Emotional Involvement (EI)		
EI1	I feel sad for this blogger when he/she makes a mistake or when bad things happen to him/her.	Kim et al. (2018)
EI2	When watching this blogger’s content about time-honoured restaurants, I felt that I was part of the story.	
EI3	When this blogger appears in the content about time-honoured restaurants, he/she is like an old friend of mine.	
EI4	After having watched this blogger’s content about time-honoured restaurants, I still thought about this blogger.	
EI5	When watching this blogger’s content about time-honoured restaurants, I feel comfortable as if he/she was my friend.	
Behavioural Intention (BI)		
BI1	I would go to this time-honoured restaurant for consumption in the future if I have the opportunity.	Liu et al. (2005), Lin (2006), Gu et al. (2021)
BI2	I expect to go to this time-honoured restaurant for consumption in the future.	
BI3	I would like to recommend this time-honoured restaurant to whoever is interested in time-honoured restaurants.	
BI4	I would like to leave a relatively positive comment on this time-honoured restaurant on social media.	
BI5	I plan to go to this time-honoured restaurant for consumption in the future.	
BI6	I would recommend this time-honoured restaurant to my friends and family.	

### Analytical procedure

Firstly, the data were initially processed and descriptively analysed by SPSS software, and then the structural equation model was evaluated by partial least squares (PLS-SEM) using SmartPLS software. A PLS-SEM approach was selected owing to its proven effectiveness in analysing exploratory research models (Hair et al., 2011). As our study aimed to explore how nostalgia affects behavioural intentions, PLS-SEM was the most appropriate method. The analysis process was divided into two parts, according to Hair et al. (2011), which included evaluating both the measurement and structure models.

## Results

### Sampling profiles

This research survey was completed by 330 individuals, of which 319 were valid. Guangzhou Restaurant (26.33%), Tao Tao Ju (23.51%), Yin Ji (6.90%), Lian Xiang Lou (5.96%) and Xiang Qun Restaurant (3.45%) were the top five brands mentioned in the survey. Table 2 provides demographic information derived from the formal investigation stage regarding respondents. Male and female respondents accounted for 38.87 and 61.13%, respectively. For the age group, most respondents were between the ages of 18 and 35. Regarding education and income level, respondents with a bachelor’s degree accounted for 43.89%, and the majority had a monthly income of 10,000 Yuan or less. The number of times respondents viewed relevant bloggers’ content per week was pretty evenly distributed, with 25.08% twice a week, 28.84% thrice a week and 21.32% five times or more each week. According to the suggestion of Memon et al. (2020), it is recommended that the ratio of samples to items should be no less than five to one. There are 32 items in this research questionnaire, so obtaining 319 respondents meets the minimum sample size requirements.

**Table 2:** Respondent demographics.

Characteristics	*N* (319)	Percentage
**Gender**		
Male	124	38.87%
Female	195	61.13%
**Age**		
18–25	143	44.83%
26–35	124	38.87%
36–45	34	10.66%
46–55	13	4.07%
56–65	5	1.57%
66 or above	0	0.00%
**Education**		
High school or below	76	23.82%
Diploma	91	28.53%
Undergraduates	140	43.89%
Graduates or above	12	3.76%
**Monthly Income**		
RMB 5,000 or below	127	39.81%
RMB 5,001–10,000	138	43.26%
RMB 10,001–20,000	37	11.60%
RMB 20,001 or above	17	5.33%
**Weekly frequency**		
One time	56	17.55%
Two times	80	25.08%
Three times	92	28.84%
Four times	23	7.21%
Five times or above	68	21.32%
**The time-honoured brand mentioned**		
Guangzhou Restaurant	84	26.33%
Tao Tao Ju	75	23.51%
Yin Ji	22	6.90%
Lian Xiang Lou	19	5.96%
Xiang Qun Restaurant	11	3.45%
Other time-honoured brands	68	33.85%

### Measurement model

A measure of the reliability of a measurement model is determined by Cronbach’s α and composite reliability. In Table 3, the values of Cronbach’s α ranged from 0.882 to 0.955, and the CR value ranged from 0.911 to 0.962. Consequently, the measurement model is reliable as both indicators are greater than 0.7 (Hair et al., 2019). Another two indicators, namely, factor loadings and AVE, were used to assess if the measurement model has acceptable convergent validity. Table 3 shows the satisfactory results. Values of factor loading ranging from 0.771 to 0.916 and AVE ranging from 0.630 to 0.781 were above the threshold (Hair et al., 2019).

**Table 3:** Reliability and convergent validity.

Construct	Items	Factor loading	Cronbach’s alpha	Composite reliability	AVE
Nostalgia (NOS)			0.955	0.962	0.737
	NOS1	0.792			
	NOS2	0.868			
	NOS3	0.896			
	NOS4	0.870			
	NOS5	0.888			
	NOS6	0.835			
	NOS7	0.882			
	NOS8	0.779			
	NOS9	0.906			
Parasocial interaction (PSI)			0.889	0.915	0.643
	PSI1	0.793			
	PSI2	0.832			
	PSI3	0.811			
	PSI4	0.771			
	PSI5	0.799			
	PSI6	0.805			
Credibility (CRED)			0.882	0.911	0.630
	CRED1	0.837			
	CRED2	0.831			
	CRED3	0.796			
	CRED4	0.767			
	CRED5	0.766			
	CRED6	0.762			
Emotional involvement (EI)			0.916	0.937	0.749
	EI1	0.801			
	EI2	0.845			
	EI3	0.899			
	EI4	0.862			
	EI5	0.916			
Behavioural intention (BI)			0.944	0.955	0.781
	BI1	0.892			
	BI2	0.882			
	BI3	0.880			
	BI4	0.848			
	BI5	0.907			
	BI6	0.894			

A Fornell-Larcker criterion was used in conjunction with a HTMT analysis to determine the acceptable criteria for discriminant validity. In Table 4, a satisfying result of the Fornell–Larcker criterion could be confirmed because the square root of the AVE shown in the bold font was greater than the correlations of each construct shown below the bold fonts. For the HTMT analysis, most of the HTMT values were less than 0.85, and only the value between parasocial interaction and emotional involvement was 0.873, which was still less than 0.9; hence, HTMT analysis still had an acceptable result (Hair et al., 2022).

**Table 4:** Discriminant validity.

Construct	NOS	PSI	CRED	EI	BI
Nostalgia (NOS)	**0.858**	0.729	0.633	0.725	0.695
Parasocial Interaction (PSI)	0.674	**0.802**	0.777	0.873	0.725
Credibility (CRED)	0.586	0.696	**0.794**	0.739	0.696
Emotional Involvement (EI)	0.677	0.789	0.670	**0.865**	0.703
Behavioural Intention (BI)	0.661	0.670	0.638	0.657	**0.884**

The square root of the AVE (the bold fonts); HTMT ratios (above the bold fonts); Correlations (below the bold fonts).

**Figure 2:**
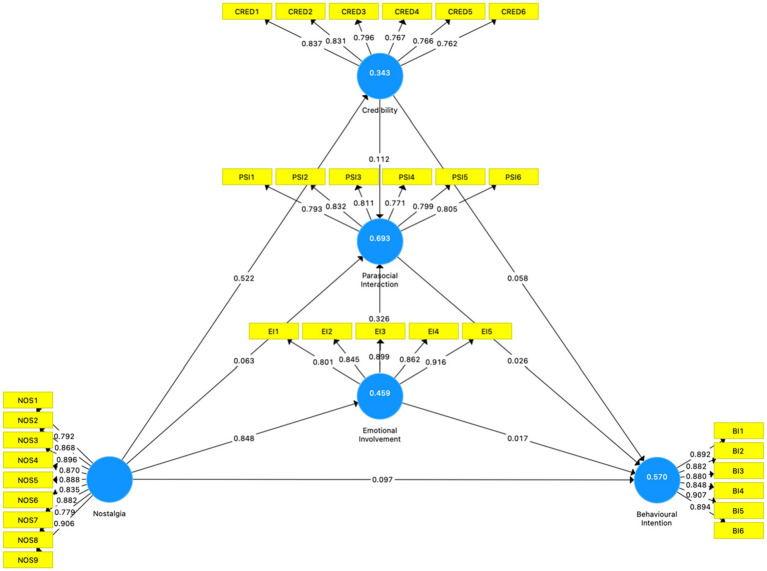
PLS Algorithm in SmartPLS.

The PLS algorithm calculated the above data results. Figure 2 is the output picture in SmartPLS software showing the results of the PLS algorithm. The data between the yellow box and the blue circle is the factor loading of each item. R^2^ (the data on the blue circles) and ƒ^2^ (the data between the blue circles) will be used in the structural model evaluation.

### Structural model

In the structural model step, the values of R^2^ and Q^2^ were initially used to evaluate if the structural model was capable of accurately predicting the data. The minimum acceptable values for R^2^ and Q^2^ are 0.1 and 0, respectively (Falk and Miller, 1992; Hair et al., 2022). PLS algorithm calculated the value of R^2^, and the value of Q^2^ was obtained by Blindfolding. Table 5 shows that R^2^ values ranging from 0.343 to 0.693, and Q^2^ values ranging from 0.21 to 0.438, establishing the acceptable predictive capability of the structural model.

**Table 5:** Determination coefficient and predictive correlation.

Construct	R Square	Q Square
Parasocial interaction	0.693	0.438
Credibility	0.343	0.210
Emotional involvement	0.459	0.340
Behavioural intention	0.570	0.434

Owing to the self-report nature of the data as well as the fact that the data were collected only once, bias is a possibility which results from common method bias. To diagnose the bias, a complete collinearity assessment approach was employed as recommended by Kock (2015). Table 6 shows that all variance inflation factors (VIF) ranging from 1 to 3.255 were less than 3.3, establishing that the common method bias was not present in this study’s research model. As a rule of thumb, considering that none of the VIFs exceeded 5, multicollinearity did not pose a barrier to this study.

**Table 6:** Results of VIF, effect size, and hypothesis analysis.

Hypothesis	Coefficient	*T*-statistics	*p*-values	ƒ^2^	VIF	Result
H1. Nostalgia → Parasocial Interaction						
	0.195	3.039	0.002**	0.063	1.962	Accept
H2. Nostalgia → Credibility						
	0.586	13.632	0.000***	0.522	1.000	Accept
H3. Nostalgia → Emotional Involvement						
	0.677	18.066	0.000***	0.848	1.000	Accept
H4. Credibility → Parasocial Interaction						
	0.258	3.449	0.001**	0.112	1.928	Accept
H5. Emotional Involvement → Parasocial Interaction						
	0.484	6.528	0.000***	0.326	2.341	Accept
H6. Credibility → Behavioural Intention						
	0.231	3.388	0.001**	0.058	2.144	Accept
H7. Emotional Involvement → Behavioural Intention						
	0.152	1.828	0.064	0.017	3.103	Reject
H8. Parasocial interaction → Behavioural Intention						
	0.191	2.344	0.020*	0.026	3.255	Accept
H9. Nostalgia → Behavioural Intention						
	0.295	4.392	0.000***	0.097	2.086	Accept

*** *p* < 0.001; ** *p* < 0.01; * *p* < 0.05.

**Figure 3:**
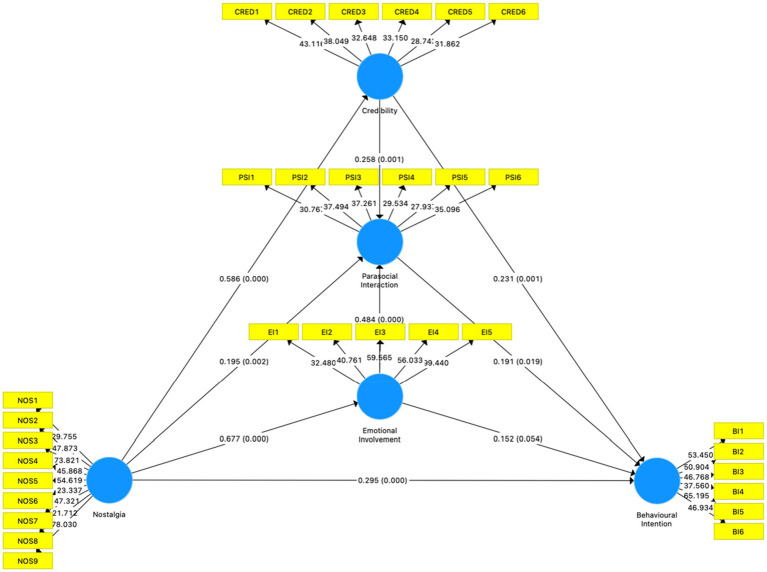
Bootstrapping with 5,000 Samples in SmartPLS.

Afterwards, the statistical significance between constructs was tested using the bootstrapping method with 5,000 resamples. Figure 3 is the result of Bootstrapping with 5,000 samples in SmartPLS. The specific data are shown in Table 6.

As illustrated in Table 6, nostalgia has a positive effect on parasocial interaction (*β* = 0.195, *p* < 0.01), credibility (*β* = 0.586, *p* < 0.001) and emotional involvement (*β* = 0.677, *p* < 0.001), thus supporting H1 to H3; parasocial interaction can be significant as predicted by credibility (*β* = 0.258, *p* < 0.01) and emotional involvement (*β* = 0.484, *p* < 0.001), thus supporting H4 and H5; behavioural intention can be significant as predicted by credibility (*β* = 0.231, *p* < 0.01), parasocial interaction (*β* = 0.191, *p* < 0.05) and nostalgia (*β* = 0.295, *p* < 0.001) but not by emotional involvement (*β* = 0.152, *p* > 0.05). The results supported H6, H8 and H9, but not H7.

The ƒ^2^ values are also used to determine the effect size as part of the hypothesis quality testing process. As can be seen from Table 6, except for hypothesis 7, the ƒ^2^ values of the remaining hypotheses are all greater than 0.02, indicating that each significant hypothesis has acceptable quality (Hair et al., 2022).

To identify the reason why H7 cannot be verified, the bootstrapping resampling method (5,000 resamples) was also applied to confirm the statistical significance of the indirect path. Table 7 demonstrates nine indirect paths in the research model. The significant relationship of indirect path (IP) 8 (β = 0.092, p < 0.05) proved that H7 was not valid as parasocial interaction entirely mediated between emotional involvement and behavioural intention. In addition, comparing the accepted result of IP2 and the rejected result of IP5 demonstrated the different effects that emotional involvement and credibility have on behavioural intention through parasocial interaction, respectively. Further discussion of the theoretical and practical implications of indirect path analysis will be provided in the following section.

**Table 7:** Results of indirect path analysis.

Indirect Path	Coefficient	*T*-statistics	*p*-values	Result
IP1. Nostalgia → Parasocial interaction → Behavioural Intention				
	0.037	2.043	0.041*	Accept
IP2. Nostalgia → Emotional Involvement → Parasocial Interaction → Behavioural Intention				
	0.063	2.022	0.043*	Accept
IP3. Nostalgia → Emotional Involvement → Parasocial Interaction				
	0.328	5.968	0.000***	Accept
IP4. Nostalgia → Emotional Involvement → Behavioural Intention				
	0.103	1.933	0.053	Reject
IP5. Nostalgia → Credibility → Parasocial Interaction → Behavioural Intention				
	0.029	1.719	0.086	Reject
IP6. Nostalgia → Credibility → Parasocial Interaction				
	0.151	3.280	0.001**	Accept
IP7. Nostalgia → Credibility → Behavioural Intention				
	0.135	3.376	0.001**	Accept
IP8. Emotional Involvement → Parasocial Interaction → Behavioural Intention				
	0.092	2.088	0.037*	Accept
IP9. Credibility → Parasocial interaction → Behavioural Intention				
	0.049	1.787	0.074	Reject

*** *p* < 0.001; ** *p* < 0.01; * *p* < 0.05.

## Discussion

The current study demonstrates how the stimulus evoked by blogger’s content influences viewers’ psychological states and processes. The results confirm H2 and H3, indicating that the more nostalgia blogger’s content evokes, the more credible the viewers perceive the blogger to be (H2), and the more emotional involvement with the blogger they generate (H3). The current study necessarily explores the relationship between nostalgia and parasocial interaction. The result crucially affirms the important role of nostalgia evoked by blogger’s content, which positively influences parasocial interaction between viewers and the blogger (H1).

As a variable intermediate between the organism (O) and the response (R) in the SOR model, parasocial interaction is also affected by credibility and emotional involvement. According to the results, H4 and H5 are supported, respectively, indicating that both credibility of the blogger (H4) and viewers’ emotional involvement (H5) can further foster the parasocial interaction between viewers and the blogger, which is consistent with previous studies (Zhang et al., 2020; Yılmazdoğan et al., 2021; Zheng et al., 2022).

The current study has proposed four hypotheses (H6 to H9) to investigate what factors would and how to influence behavioural intention. The results show that H6, H8and H9 are all supported, whereas H7 is rejected. Specifically, viewers’ positive behavioural intentions are affected by the high level of blogger credibility (H6), high level of parasocial interaction between the blogger and viewers (H8) and high number of nostalgia triggers the blogger’s content evokes (H9). The supported results are consistent with previous research (Chen et al., 2014; Gong and Li, 2017; Saima and Khan, 2020). H7 is rejected probably be due to the fact that viewers’ emotional involvement with the blogger is based on specific content, and this emotional involvement may be motivated by the enjoyment of the entertainment and immersion in the blogger’s content, which does not directly result in restaurant consumption intention. Interestingly, we found a full mediating role played by parasocial interaction between emotional involvement and behavioural intention (IP8), that is, viewers’ emotional involvement with the blogger positively relates to behavioural intention by enhancing parasocial interaction. Moreover, to generate behavioural intentions related to restaurant consumption, viewers need to transform their emotional involvement with the blogger in a particular content into parasocial interaction with more motivational potential.

In addition, according to the accepted results of IP3 and IP6, nostalgia could indirectly foster parasocial interaction by enhancing both credibility and emotional involvement. However, the result of IP2 is accepted, but that of IP5 is rejected. Thus, the emotional involvement affected by nostalgia contributes more benefits than the credibility affected by nostalgia on the path to enhancing behavioural intention by fostering parasocial interaction.

## Conclusion and implications

Based on the SOR model, this study establishes a comprehensive framework for nostalgia marketing of time-honoured restaurants using bloggers’ content as a carrier, thereby enriching the theory of nostalgia marketing in the context of online media and expanding the application scenarios of parasocial interaction theory and SOR theory in the field of time-honoured restaurants marketing. In addition, the results of this study could provide some strategic ideas and help for the marketing of time-honoured restaurants in the present era.

### Theoretical implications

In theory, from a cross-disciplinary perspective, this study combines the knowledge of psychology, communication and marketing disciplines to establish a theoretical framework from nostalgic stimuli to parasocial interaction and thus to behavioural intention, providing theoretical support for a marketing model that integrates tradition and innovation.

Most of the past studies on nostalgia have treated nostalgia as a direct stimulus of the live environment to respondents (Hwang and Hyun, 2013; Chen et al., 2014; Song et al., 2021). This study successfully explores the important potential of nostalgia in the context of bloggers’ content and provides a new research vision for nostalgia marketing. Accordingly, the parasocial interaction was investigated on the basis of the group of bloggers who generate content about time-honoured restaurants and their viewers, thus expanding the scope of the application of parasocial interaction.

Most importantly, to the best of our knowledge, the current study is the first to establish a link between nostalgia evoked by bloggers’ content and parasocial interactions. The findings suggest that this nostalgia fosters parasocial interactions between viewers and bloggers, thereby enriching the existing predictors of parasocial interaction. In this way, nostalgia can be presented in a new form. Indeed, part of the online media content could be seen as a huge attic or bric-a-brac market where individual and collective nostalgias converge and spread (Niemeyer, 2014). A profitable return on nostalgic products must reflect modern nostalgia, and the ‘old’ and the ‘new’ must complement each other (Cui, 2015). Our study further verifies the relationship between old and new, highlighting the balance between nostalgic tradition and innovative display form. An approach for retro-innovation that uses a new format to meet an old need may get easier to attract consumers and enhance economic sustainability (León-Bravo et al., 2019). The findings of the current study may provide some support for research that focuses on such types of marketing.

To explore in further detail how nostalgia affects parasocial interaction and ultimately influences behavioural intention, the current study incorporates credibility and emotional involvement into the organism (O) as cognitive and affective states, respectively, which is consistent with the description that the organism has both states (Eroglu et al., 2001) and somewhat ensures the integrity of organism (O). This study demonstrates that both credibility and emotional involvement as organisms are affected by nostalgia as the stimulus. However, credibility directly and positively influences behavioural intention, whilst emotional involvement indirectly enhances behavioural intention through the full mediating role of parasocial interaction. This finding is consistent with the situation that the cognitive and affective influences on response (R) vary across different types of restaurant contexts (Kim and Moon, 2009; Demoulin, 2011; Leung et al., 2021). According to the viewpoint of Leung et al. (2021), this difference may be associated with the different values (e.g., hedonic and utilitarian) that customers pursue across various contexts; for quick-service restaurants, customers primarily pursue the utilitarian value of satisfying hunger and efficiency; thus, the process from stimulus (S) to response (R) is likely to focus on cognition rather than emotion. Consequently, the following can be inferred from the above propositions: for the slow-paced type of restaurants such as time-honoured restaurants, customers should pursue more hedonic values such as restaurant ambiance and the enjoyment of dining, and the process from stimulus (S) to response (R) should focus more on emotion rather than cognition. However, in the context of nostalgia stimuli evoked by bloggers’ content, emotional involvement does not directly influence behavioural intention but through parasocial interaction, whilst credibility can directly influence behavioural intention. In other words, if we regard the situation from the perspective of influencing behavioural intention through parasocial interaction, the contribution of emotional involvement is stronger than credibility, which is consistent with the above viewpoint of Leung et al. (2021).

### Practical implications

In practice, this study contributes to two main parts of restaurant marketing and blogger operations, with the results suggesting several pathways to increase the parasocial interaction between viewers and bloggers and the behavioural intention of customers or potential customers.

Firstly, nostalgia evoked by bloggers’ content is demonstrated to directly enhance parasocial interaction and behavioural intention. For nostalgia-themed related restaurants that use blogger content marketing, apart from developing nostalgia triggers in a realistic environment (e.g., food, indoor environments, the old style of service) (Song et al., 2021), attaching importance to the construction of atmosphere in the bloggers’ content is feasible. For example, bloggers could wear vintage clothing and tell stories in a slow, memory-evoking style. In addition, traditional music and vintage ambient tones are also recommended to establish a nostalgic atmosphere. In this way, the blogger can foster parasocial interaction with viewers, and the restaurant can attract customers. We believe that nostalgia is not only an attribute based on the product itself, it can also be reflected in bloggers’ content and can strengthen the parasocial interaction between the viewers and the bloggers. The nostalgia of the product itself may only target a specific group of people, whereas the nostalgia evoked through bloggers’ content can attract larger potential consumers. This strategy of incorporating digital marketing intelligence innovation into time-honoured brands is valuable (Huang, 2022) and can attract more young people to the point where time-honoured restaurant consumers can cover a wider age range. Meanwhile, through the relevant contents communicated by bloggers, the cultural value of time-honoured restaurants is efficiently extracted and presented to the viewers, who can more directly feel the warmth and significance of time-honoured restaurants in modern aesthetics. Consequently, potential consumers can be tapped for time-honoured restaurants. Additionally, by building an emotional atmosphere, people’s cognition on the beauty of culture can be awakened in their hearts, thus indirectly promoting the retention and inheritance of traditional culture.

Secondly, both cognitive and affective factors need consideration in the restaurant business or blogger operations. From a cognitive perspective, improving the credibility of bloggers can directly contribute to the behavioural intentions of viewers, thus resulting in positive word-of-mouth (WOM) or electronic word-of-mouth (e-WOM) and consumer behaviour. Therefore, selecting a blogger with high credibility is needed to act as a promoting ambassador. On the other hand, a blogger who has a high level of credibility can easily build a favourable relationship or create further interactions with the viewers. Bloggers should specialise in their fields, prioritise trustworthiness and generate honest and credible content to foster parasocial interaction with viewers (Yuan and Lou, 2020). From an affective perspective, extra attention should be paid to increasing the emotional involvement of bloggers’ content viewers and forming the stable parasocial interaction that is not limited to being inspired a specific content. For example, paying extra attention to the content presentation from the viewer’s perspective to achieve empathy is worth considering. From a realistic perspective, There is no absolute answer to the question of which factor is more crucial, cognitive or affective. Numerous factors need to be taken into account, including the specific type of restaurant (Kim and Moon, 2009), whilst comprehensive consideration is better for bloggers.

Thirdly, we confirm that nostalgia can indirectly enhance behavioural intention through credibility but not emotional involvement. When watching blogger’s content, if the viewers perceive the time-honoured restaurant as merely an old thing and fail to feel a sincere desire for nostalgia within them, or even feel forced to be nostalgic for something untrustworthy, then they will conversely be more antipathetic to such things. Therefore, time-honoured restaurant managers need to utilize nostalgia to achieve their marketing purposes, more specifically, authentically restore the original appearance of the restaurant and relevant information, and demonstrate their dedication to passing on traditional cuisine, thereby conveying the warmth and honesty of the business.

Fourthly, relevant policy support is also critical in order to solve the survival plight of time-honoured restaurants. Local departments can organise online activities that evoke a sense of nostalgia for traditional food culture in the community to cultivate a vibrant cultural atmosphere. At the same time, the government can introduce supportive policies in light of local specificities. For example, providing a well-suited online promotion platform for time-honoured restaurants, inviting celebrities to promote them and bring them into the lives of the public, or even combining the food culture of time-honoured restaurants with local tourism culture, city image and other distinctive elements, so as to carry out diversified publicity. In addition, attention should be paid to the supervision of online platform marketing and a strict crackdown on information falsification to prevent damage to the image and reputation of time-honoured restaurants.

## Limitations and future studies

Following the discussion and analysis of the research process and results, we conclude that this study has some limitations, and we also propose some suggestions and ideas for future research.

Firstly, this study took the form of a questionnaire to test all hypotheses, which may be difficult to ensure that other sources of information interfere with the assessment of behavioural intentions. Future research could employ experimental methods to test whether people will have behavioural intentions towards those designated unfamiliar time-honoured restaurant as a result of a blogger’s promotion in the form of nostalgia. Secondly, considering that parasocial interaction may be a dynamic perceptual state, the cross-sectional data in this study may somewhat lack accuracy in reflecting the true state of the viewers. Therefore, using a longitudinal study is worthwhile to explore parasocial interactions for further detail in the context of online marketing. Thirdly, we left the results of nostalgia caused by the bloggers’ content at the level of behavioural intention. Future research could further explore the actual behaviour of viewers. Fourthly, this study adopts an online survey and quantitative research approach based on the context of Chinese cities. Future research could visit time-honoured restaurants to collect data on site, adopt a qualitative research approach or explore the results in different geographical contexts from a cross-cultural perspective to enrich the relevant theoretical framework and research insights. In addition, various types of nostalgic stimuli are shown in the blogger’s content. Thus, examining the differences in the consequences caused by specific different types of nostalgic stimuli is worthy of consideration.

## Data availability statement

The raw data supporting the conclusions of this article will be made available by the authors, without undue reservation.

## Ethics statement

Ethical review and approval was not required for the study on human participants in accordance with the local legislation and institutional requirements. Written informed consent for participation was not required for this study in accordance with the national legislation and the institutional requirements.

## Author contributions

JY and JT: conceptualization, data curation, formal analysis, investigation, methodology, visualization, and writing-original draft. LZ: supervision and project administration. JY: validation. JT, JY, and LZ: writing-review and editing. All authors contributed to the article and approved the submitted version.

## Funding

Guangdong Provincial Philosophy and Social Sciences Lingnan Culture Project, GD21LN14.

## Conflict of interest

The authors declare that the research was conducted in the absence of any commercial or financial relationships that could be construed as a potential conflict of interest.

## Publisher’s note

All claims expressed in this article are solely those of the authors and do not necessarily represent those of their affiliated organizations, or those of the publisher, the editors and the reviewers. Any product that may be evaluated in this article, or claim that may be made by its manufacturer, is not guaranteed or endorsed by the publisher.
